# Safety and Feasibility of Chemoembolization with Doxorubicin-Loaded Small Calibrated Microspheres in Patients with Hepatocellular Carcinoma: Results of the MIRACLE I Prospective Multicenter Study

**DOI:** 10.1007/s00270-017-1839-2

**Published:** 2017-11-22

**Authors:** Götz Richter, Boris Radeleff, Christian Stroszczynski, Philippe Pereira, Thomas Helmberger, Mark Barakat, Peter Huppert

**Affiliations:** 1 1st Siemens Reference Center for Interventional Radiology and Oncology, Klinikum Stuttgart, Kriegsbergstr 60, 70174 Stuttgart, Germany; 2 Klinikum der Universität Heidelberg, Heidelberg, Germany; 3 Klinikum der Universität Regensburg, Regensburg, Germany; 4 SLK-Kliniken Heilbronn GmbH, Heilbronn, Germany; 5 Klinikum Bogenhausen, Munich, Germany; 6 Celonova Biosciences, San Antonio, TX USA; 7 Klinikum Darmstadt, Darmstadt, Germany

**Keywords:** Chemoembolization, Doxorubicin, Drug carriers, Hepatocellular carcinoma, Microspheres

## Abstract

**Purpose:**

The MIRACLE I pilot study was designed as a preliminary investigation of safety and efficacy of Embozene TANDEM microspheres loaded with doxorubicin for treatment of locally untreatable (i.e., unresectable and not suitable for local thermal ablation) hepatocellular carcinoma (HCC).

**Materials and Methods:**

Patients with locally untreatable HCC (mono- or bilobar disease, ECOG performance status 0–2, Child–Pugh score < 11) were eligible for this single-arm multicenter study. DEB-TACE was performed with 75 µm Embozene TANDEM loaded with 150 mg of doxorubicin.

**Results:**

Twenty-five subjects with 41 tumors were treated (mean age 65 years); 16, 52, and 32% had BCLC A, B, and C status, respectively. Child–Pugh status was A for 64%, B for 32%, and C for 4%; 40% had ascites. About 92% had disease localized to one liver lobe. Most (72%) underwent ≤ 2 DEB-TACE procedures. Average doxorubicin dose was 124.5 ± 36.1 mg (median 150 mg) per procedure. Two patients had procedure-related SAE (liver necrosis, worsening of liver insufficiency) within 30 days of the first DEB-TACE procedure. Six-month freedom from procedure-related SAE or death was 68% (one hepatic encephalopathy, five deaths). Tumor response or stable disease was achieved in 95% (20/21) of subjects. Freedom from tumor progression or death at 6 months was 76%. The one-year survival rate was 56% overall and 73% among patients without ascites at baseline.

**Conclusion:**

MIRACLE I results suggest that Embozene TANDEM microspheres loaded with doxorubicin can provide good local tumor control in a heterogeneous group of patients with locally untreatable HCC.

**Level of Evidence:**

Level 2b, Individual cohort study.

## Introduction

Patients with locally untreatable HCC (i.e., unresectable and not suitable for local thermal ablation) have few treatment options [1]. Systemic sorafenib has been shown to modestly prolong survival in patients with advanced stage disease [1, 2], and transarterial chemoembolization (TACE) is recommended for patients with intermediate stage disease [1]. Meta-analyses have shown that TACE performed with doxorubicin-loaded beads has similar efficacy but fewer side effects than conventional TACE [3, 4].

In a preliminary single-center study of 52 patients with locally untreatable HCC who underwent TACE with doxorubicin-loaded Embozene TANDEM™ microspheres (CeloNova Biosciences/Boston Scientific, Marlborough, MA), more than 60% of patients had an objective tumor response, and the one-year survival rate was more than 90% [5]. All patients in the study were treated with microspheres of nominal diameter ≤ 100 µm and were randomized to one of two doxorubicin doses. The MIRACLE I pilot study was a multicenter study designed to further evaluate safety and efficacy of Embozene TANDEM microspheres loaded with doxorubicin for DEB-TACE treatment of locally untreatable hepatocellular carcinoma, specifically the 75 µm size and 150 mg doxorubicin dose.

## Materials and Methods

### Study Design

Microparticle Enhanced Cytotoxic Transarterial Embolization Therapy in Hepatocellular Carcinoma (MIRACLE I) was a prospective, single-arm multicenter feasibility study of chemoembolization with 75 µm doxorubicin-loaded Embozene TANDEM microspheres to treat locally unresectable HCC.

The trial was conducted according to the guidelines established in the Declaration of Helsinki, ICH/135/95 Good Clinical Practice (GCP), and local ethical and legal requirements. Ethics committee and competent authority approvals were obtained prior to study initiation. All patients provided written informed consent. The trial was registered at clinicaltrials.gov (identifier NCT01798134).

### Study Patients

Patients eligible for the study were adults with confirmed diagnosis of HCC according to European Association of the Study of the Liver (EASL) criteria [6] and were staged according to BCLC criteria [1]. Eastern Cooperative Oncology Group (ECOG) [7] performance status of 0, 1, or 2, and Child–Pugh score of < 11 points (i.e., A, B, or C 10) [8] were required. Tumors were to be ≥ 3–10 cm (multinodular or single node). Mono- or bilobar disease was allowed. Lack of main portal vein trunk or common bile duct invasion was to be confirmed by MRI. Patients were required to have laboratory values in the following ranges: white blood cell count > 3000/mL, absolute neutrophil count > 1500 cells/mL, INR < 2.0, partial thromboplastin time < 40 s, platelet number > 5 × 10^4^ mL, blood bilirubin < 3.0 mg/dL, aspartate aminotransferase (AST), and alanine aminotransferase (ALT) within five times of normal range of each organ, serum creatinine < 2.5 mg/dL, hemoglobin > 8.0 g/dL, and alkaline phosphatase < 630 IU/L. Key exclusion criteria were metastatic disease or diffuse HCC, the presence of an untreatable arteriovenous or arterioportal shunt, known hepatofugal blood flow, previous treatment with anthracyclines, previous embolotherapy to treat primary liver cancer, and treatment with sorafenib within the previous 4 weeks. Patients with unstable coronary artery disease, myocardial infarction within the previous 4 weeks, or an abnormal electrocardiogram (QT < 480 ms) within the previous 12 months were excluded.

### Treatment Plan and Embolization Procedure

Embozene TANDEM microspheres have a negatively charged hydrogel core and biocompatible perfluorinated polymer coating. They can be loaded with anthracyclines, such as doxorubicin, or other chemotherapeutic drugs, such as irinotecan [5, 9–11]. Patients in MIRACLE I were treated with 75 µm diameter Embozene TANDEM microspheres, which are calibrated to ± 15 µm. One 3-mL syringe of microspheres can be loaded with up to 150 mg of doxorubicin-HCl, with little shrinkage (~ 9%) observed after drug loading [9]. Doxorubicin loading was performed according to the manufacturer’s instructions.

A procedural doxorubicin dose of 75 mg/m^2^ body surface area was targeted. A minimum of two treatments per lesion, separated by 4 weeks, was planned. Thus, patients with bilobar hepatic disease were to have at least four treatment sessions (two per liver lobe).

Chemoembolization procedures were performed with antibiotic prophylaxis, analgesic, and antiemetic medications at the physician’s discretion. Angiography of the hepatic and mesenteric arteries was performed prior to chemoembolization to confirm anatomical eligibility and identify tumor feeder arteries. The hepatic segmental or subsegmental arteries supplying the lesion were selectively catheterized with a microcatheter while ensuring sufficient flow to the tumor, and a mixture of 75-µm doxorubicin-loaded microspheres and non-ionic contrast agent was slowly injected. The protocol encouraged a super-selective approach to reduce non-target embolization. Bland microspheres could also be used at the treating physician’s discretion if blood flow stasis was not achieved after delivery of the desired drug dose.

### Safety and Efficacy Endpoints

The primary study endpoints were safety (serious adverse events) at 30 days and 6 months, and freedom from primary tumor progression at 6 months. Secondary endpoints included the rate of local tumor control and 12-month survival. Survival among subgroups of patients with Child–Pugh class A or B/C liver function and with or without ascites was examined in a post hoc analysis.

Adverse event monitoring occurred throughout the treatment and follow-up phases. Adverse event seriousness and potential causal relationships with the DEB-TACE procedure or Embozene TANDEM microspheres were assessed by the site investigator. Grading according to CTCAE criteria was not mandated in the study protocol, but was completed retrospectively (CTCAE v 4.03) for serious adverse events occurring within 30 days post-procedure based on information collected about the events.

Tumor imaging (contrast-enhanced CT or MRI) was performed, and measurements were taken within 2 weeks prior to the first DEB-TACE procedure (baseline) and 2 weeks following the first and again following the second procedure (for a patient with monolobar disease intended to be treated with a minimum of two procedures). Repeat DEB-TACE procedures were performed when follow-up imaging studies showed residual enhancement and patients continued to be willing to undergo additional procedures. Imaging was repeated at 4- to 6-week intervals to determine need for additional DEB-TACE procedures and was scheduled to be performed 3 and 6 months following the last DEB-TACE procedure and 12 months from the initial treatment. Clinical and laboratory assessments were also repeated at each of these visits.

Tumor response was assessed based on mRECIST criteria [12]. “Best overall response” was defined as the smallest measurement of hypervascularized tumor tissue recorded from the start of study treatment until disease progression/recurrence and took into account non-target and new lesions [6]. Images were evaluated by the investigator/radiologist at each site.

## Results

### Patients

From January 2013 to March 2014, 25 patients with 41 lesions were enrolled and treated at six German sites. Patient and lesion characteristics are summarized in Table 1. Most patients (92%) had monolobar disease, and 32% had advanced HCC (BCLC stage C). Thirteen (52%) patients had at least one tumor with largest diameter > 5 cm and eight more patients (32%) had at least one tumor with largest diameter 3–5 cm. As shown in Table 1, Child–Pugh classification of B (Child–Pugh score of 7–9) or C (Child–Pugh score of 10) was noted for 36% of patients at baseline, and many (64%) presented with symptoms of advanced liver disease, including ascites, hepatic encephalopathy, esophageal varices, or splenomegaly. Five patients had radiofrequency ablation or surgical resection prior to enrollment; none had systemic chemotherapy. Several patients had comorbidities preventing surgery, and two were on the liver transplant list at enrollment. One patient was withdrawn from the study two days after the initial treatment in order to undergo systemic treatment outside the trial; this patient was followed for safety but was excluded from the efficacy analysis. Four other patients withdrew consent (one at 5 days, one at 2 weeks, and two at 5 months from the initial treatment), and one patient was withdrawn after suffering a fall and broken vertebrae 2 weeks following the initial treatment.

**Table 1:** Baseline patient characteristics (*N* = 25)

Characteristic	*N* (%)
Sex (male/female)	18/7
Age, years (median, range)	65 (39–85)
Race/ethnicity	
Caucasian	24 (96)
Asian	1 (4)
Etiology of cirrhosis	
Alcohol abuse	12 (48)
HCV	3 (12)
HBV	3 (12)
HBV, HCV, and alcohol abuse	1 (4)
NASH	1 (4)
Unknown/other	5 (20)
Child–Pugh classification	
A	16 (64)
B	8 (32)
C	1 (4)
ECOG performance status	
0	19 (76)
1	5 (20)
2	1 (4)
BCLC classification	
A	4 (16)
B	13 (52)
C	8 (32)
Prior radiofrequency ablation therapy	1 (4)
Prior surgery	4 (16)
INR < 1.5	24 (96)
Platelets < 100,000	7 (28)
Bilirubin ≤ 2	22 (88)
AST < 100	22 (88)
ALT < 100	25 (100)
Ascites	10 (40)
Hepatic encephalopathy	2 (8)
Esophageal varices	9 (36)
Splenomegaly	9 (36)
Portal vein thrombosis	2 (8)
Diabetes	11 (44)
Anemia	5 (20)
Renal insufficiency	4 (16)
Liver lobes involved	
1 (Monolobar HCC)	23 (92)
2 (Bilobar HCC)	2 (8)
Tumor size (largest diameter; *n* = 41)	
<3 cm	15 (37)
3 − < 5 cm	12 (29)
≥ 5 − < 10 cm	13 (32)
≥ 10 cm	1 (2)
Range	1–13 cm

*ALT* alanine aminotransferase, *AST* aspartate aminotransferase, *HBV* hepatitis B virus, *HCV* hepatitis C virus, *HCC* hepatocellular carcinoma, and *NASH* non-alcoholic steatohepatitis

### Embolization with Drug-loaded Microspheres

All enrolled patients underwent at least one DEB-TACE procedure successfully; most had one (24%) or two (48%) procedures; five (20%) had three; and two (8%) had four or more. The six patients who underwent only one procedure all had monolobar disease; the second planned procedure was not performed because of early withdrawal from the study in four cases, as described above. One of the remaining two experienced liver necrosis and underwent hemihepatectomy approximately 1 week following treatment, and the other one died approximately 2 months following the first treatment. The administered doxorubicin dose averaged 124.5 ± 36.1 mg per procedure (median 150 mg, range 44–150 mg), with a mean cumulative dose of 227.4 ± 142.1 mg (median 180 mg, range 45–600 mg).

### Safety

By 30 days from their first embolization procedure with drug-loaded microspheres, 88% (*n* = 22) of patients remained free from procedure-related serious adverse events. The two procedure-related serious adverse events (worsening of liver insufficiency and liver necrosis) were experienced by two patients. The worsening liver insufficiency was experienced by a patient while hospitalized for a lumbar vertebrae fracture following a fall which occurred 2 weeks after the first DEB-TACE procedure. The patient had a history of cirrhosis of the liver, portal hypertension, liver insufficiency, ascites, macrocytic anemia, chronic erosive gastritis, diverticulosis, alcohol abuse, cholecystolithiasis, dyspnea, leg edema, and presented with BCLC stage C HCC. During the hospitalization (1 week later), the patient’s liver function was moderately worsened and was treated with medical treatment and prolonged hospitalization. The patient recovered and was discharged 1 week later.

The second procedure-related serious adverse event was experienced by a 60-year-old man with a history of liver cirrhosis with portal hypertension, diabetes mellitus, partial portal vein thrombosis (noted on MRI prior enrollment), hypertension, bronchial asthma, and a multifocal hepatocellular carcinoma of the right liver lobe. Two days after the first DEB-TACE procedure, the patient collapsed due to low blood pressure (60/40 mmHg) and was transferred to the intensive care unit for treatment and observation. His laboratory assessments revealed electrolyte imbalance, elevation of liver enzymes, and elevation of C-reactive protein and leukocytes. CT of the abdomen and pelvis 8 days later revealed liver necrosis around the treated segments. The patient was diagnosed to have liver cell necrosis and electrolyte imbalance. He was treated with fluid replacement, antibiotics, and prophylactic anticoagulation. His spironolactone medication was stopped due to hyponatremia. Three days later, the patient laboratory assessments were back to normal, and the next day the patient had hemihepatectomy due to the necrosis. The treating investigator reported that the patient collapse and electrolyte imbalance were due to multifactorial causes, including liver cirrhosis and necrosis, loss of volume, and concomitant medication (spironolactone), while liver cell necrosis was a result of excess volume of the microspheres and patient anatomy factors that led to embolizing the tumor and the surrounding liver tissue.

No deaths or alopecia occurred within 30 days (Table 2). Occurrence of post-embolization syndrome symptoms after sequential embolization procedures is summarized in Table 3. Post-embolization syndrome events were grade 1 or 2.

**Table 2:** Serious adverse event occurrence within 6 months of the initial embolization

	30 Days N (%)^a^	6 Months N (%)
Back pain	1 (4)	–
Acute cholecystitis	1 (4)	–
Worsening liver insufficiency	1 (4)	–
Liver necrosis^b^	1 (4)	–
Hypotension	1 (4)	–
Urinary tract infection	1 (4)	–
Gastritis	1 (4)	–
Elevated creatinine	1 (4)	1 (4)
Ascites/worsening ascites	–	3 (12)
Angina	–	2 (8)
Hepatic encephalopathy	–	1 (4)
Hepatic abscess^c^	–	1 (4)
Alopecia	–	1 (4)
Sepsis	–	1 (4)
Cardiac insufficiency	–	1 (4)
Bleeding of esophageal varices	–	1 (4)
Anemia	–	1 (4)
Melena	–	1 (4)
Ulcus lower extremity	–	1 (4)
Hemiataxia	–	1 (4)
Mortality	0 (0)	5 (20)

^a^Grade 2 unless otherwise noted

^b^Grade 3 necrosis was observed in liver tissue surrounding the tumor and was not considered immediately life-threatening

^c^Grade 3 asymptomatic and noted incidentally on abdomen CT scan performed during hospitalization due to fall

**Table 3:** Occurrence of post-embolization syndrome following DEB-TACE procedures

	1st DEB-TACE (*N* = 25)	2nd DEB-TACE (*N* = 18)	3rd DEB-TACE (*N* = 7)	4th DEB-TACE (*N* = 2)
Post-embolization syndrome, *n* (%)	13 (52)	9 (50)	6 (86)	1 (50)
Abdominal pain	8 (32)	7 (39)	2 (29)	1 (50)
Nausea/vomiting	4 (16)	2 (11)	1 (14)	1 (50)
Fever	1 (4)	2 (11)	4 (57)	0 (0)

By 6 months from their first DEB-TACE procedure, 68% (*n* = 17) of patients were free from procedure-related serious adverse events or death. One additional patient had hepatic encephalopathy reported as a possible procedure-related serious adverse event, and five patients died by 6 months. One of these deaths was the patient with the worsening liver insufficiency serious adverse event at 30 days. One was a Child–Pugh class C patient who withdrew from the study after receiving only one treatment. The other three were known to be due to comorbidities: one cardiac, one 3 days after liver transplant (documented partial response prior to transplant), and one due to decompensated liver cirrhosis (documented partial response). One patient was lost to follow-up.

### Efficacy

Imaging to evaluate tumor response was available for 21 patients (of the remaining four patients, three withdrew from the study prior to having follow-up imaging performed, and one died before response was assessed). According to mRECIST criteria for best response, complete response was achieved in 10 patients (48%), partial response in 4 (19%), stable disease in 6 (29%), and progressive disease in 1 (5%). Among these 21 patients, the median time to maximum observed radiographic response was 28 days (range 10–127 days). At 6 months, 76% of patients were free from tumor progression or death. The one patient with tumor progression had three embolization procedures before progression was observed.

The one-year survival rate was 56% (14/25); 14 patients were known alive, nine died, one was lost to follow-up, and one was not included in the efficacy analysis due to early withdrawal from the study as noted above (i.e., the patient was not treated according to the protocol). A total of four of the deaths were attributable to comorbidities: In addition to the three noted above which occurred within 6 months, a later death was caused by sepsis followed by liver failure. Survival rates were higher for patients with less advanced liver disease: The one-year survival for patients with Child–Pugh A (*n* = 16) and B/C (*n* = 9) classification was 75 and 44%, respectively, and for patients without ascites (*n* = 15) or with ascites (*n* = 10), it was 73 and 50%, respectively. Four patients received liver transplants while in the study; these occurred between 4 and 11 months following the initial DEB-TACE procedure and at a minimum of 89 days following the patient’s last procedure (each of these four patients had two study procedures). Three of the four patients who received liver transplants were alive through 12 months, the fourth died soon after their transplant as described above.

## Discussion

MIRACLE I study results demonstrated a high rate of tumor control and few serious adverse events among patients treated with doxorubicin-loaded microspheres. Incidence of serious adverse events or Grade 3–4 toxicities following doxorubicin-eluting embolic therapy for HCC has generally been low in previous studies [13–15] and was comparably low in MIRACLE I. For example, Prajapati et al. [13] reported an overall adverse event rate of 30% within 30 days of embolization, with the majority of complications Grade 1–2 and no Grade 4 toxicities in a retrospective study of 121 patients. In a randomized study, approximately 24% of 93 patients treated with a drug-eluting embolic agent reported serious adverse events within 30 days, and two patients died [14]. In MIRACLE I, no deaths or systemic toxicities occurred within 30 days, and only 2 of the 25 patients had procedure-related serious adverse events. The small 75 µm microsphere size used here was not associated with specific safety concerns. One patient developed mild alopecia in the longer term, but no myelosuppression was reported. Compared with MIRACLE I, the studies noted above included greater proportions of patients with Child–Pugh class A (> 75% in two of the studies [14, 15]) and lower proportions with ascites. Ascites was observed in 40% of MIRACLE I patients at baseline, but in only 23% of patients in one of the comparable studies [13] and excluded or not present in the other comparable study samples [14, 15]. Although the heterogeneous nature of HCC patient characteristics, variable treatment regimens, and evaluation criteria across studies limit comparisons between studies, results from previous studies of doxorubicin-loaded microsphere treatment of HCC provide background for the findings observed in the MIRACLE I study.

Post-embolization syndrome, or symptoms such as abdominal pain, nausea, vomiting, and fever, has been reported in 5–100% of patients in previous studies [5, 13–17]. In a recent study of the same type of microspheres used in MIRACLE I, post-embolization syndrome occurred in 13–46% of patients across embolization sessions [5], and approximately half of patients in MIRACLE I had mild symptoms following embolization procedures.

Tumor response rates compare favorably with previous studies, with complete or partial tumor responses observed in 67% of MIRACLE I patients and only one patient (5%) displaying tumor progression during study follow-up. Objective response rates in previous studies range from 40 to 64%, with disease progression observed in 5–32% of patients [5, 14, 15]. The one-year survival rate was 56% in the current study, with a greater rate among patients with liver function classified as Child–Pugh A (75%) or without ascites (73%). Four of the nine deaths in the MIRACLE I study were known to be attributable to comorbidities rather than to disease progression. Previously reported overall one-year survival rates range from 58 to 92% [5, 15, 18], with stratifying factors such as the presence of ascites and Child–Pugh classification of B or C associated with poorer survival [13, 18, 19]. The proportions of patients with liver function classified as Child–Pugh B or C or ascites (indicative of liver cirrhosis) were relatively high in MIRACLE I compared with these previous studies (i.e., no patients with ascites or Child–Pugh C liver disease were included in the studies by Reyes et al. [15] or Malagari et al. [5]) and may explain the lower survival rate. In the study by Dhanasekaran et al. [18], in which half of patients had Child–Pugh class B or C, one-year survival for patients who underwent TACE with drug-eluting beads was 58% overall, but decreased to 32% among patients with Child–Pugh class C.

Specific reasons for choosing to treat the study patients with TACE with drug-eluting microspheres, rather than ablation or surgery, were not collected. The presence of severe comorbidities, failed prior treatment, and multiple tumors likely limited the patients’ surgical options, thereby characterizing their disease as “locally untreatable” as defined by the study eligibility criteria. Limitations also included the lack of central review of tumor response and adverse events and the lack of systematic CTCAE grading at the time of data collection.

Notwithstanding study limitations including a relatively small sample size and heterogeneous clinical characteristics of the study sample, MIRACLE I study results suggest that Embozene TANDEM microspheres loaded with doxorubicin can provide good local tumor control in a heterogeneous group of patients with unresectable intermediate and locally advanced HCC.
